# Prognostic factors for patients with hepatic metastases from breast cancer

**DOI:** 10.1038/sj.bjc.6601038

**Published:** 2003-07-15

**Authors:** L Wyld, E Gutteridge, S E Pinder, J J James, S Y Chan, K L Cheung, J F R Robertson, A J Evans

**Affiliations:** 1 Department of Surgical and Anaesthetic Sciences, University of Sheffield, Sheffield S10 2JF, UK; 2 Department of Surgery, Nottingham City Hospital, Hucknall Road, Nottingham NG5 1PB, UK; 3 Department of Histopathology, Nottingham City Hospital, Hucknall Road, Nottingham NG5 1PB, UK; 4 Department of Radiology, Nottingham City Hospital, Hucknall Road, Nottingham NG5 1PB, UK; 5 Department of Clinical Oncology, Nottingham City Hospital, Hucknall Road, Nottingham NG5 1PB, UK

**Keywords:** breast cancer, liver metastases, prognostic variables

## Abstract

Median survival from liver metastases secondary to breast cancer is only a few months, with very rare 5-year survival. This study reviewed 145 patients with liver metastases from breast cancer to determine factors that may influence survival. Data were analysed using Kaplan–Meier survival curves, univariate and multivariate analysis. Median survival was 4.23 months (range 0.16–51), with a 27.6% 1-year survival. Factors that significantly predicted a poor prognosis on univariate analysis included symptomatic liver disease, deranged liver function tests, the presence of ascites, histological grade 3 disease at primary presentation, advanced age, oestrogen receptor (ER) negative tumours, carcinoembryonic antigen of over 1000 ng ml^−1^ and multiple *vs* single liver metastases. Response to treatment was also a significant predictor of survival with patients responding to chemo- or endocrine therapy surviving for a median of 13 and 13.9 months, respectively. Multivariate analysis of pretreatment variables identified a low albumin, advanced age and ER negativity as independent predictors of poor survival. The time interval between primary and metastatic disease, metastases at extrahepatic sites, histological subtype and nodal stage at primary presentation did not predict prognosis. Awareness of the prognostic implications of the above factors may assist in selecting the most appropriate treatment for these patients.

Liver metastases are found in 6–25% of patients with metastatic breast cancer (Tampellini *et al*, 1997). Most authors quote a median survival of 1–14 months (Jaffe *et al*, 1968; Zinser *et al*, 1987; Patanaphan *et al*, 1988; O'Reilly *et al*, 1990; Hoe *et al*, 1991) which compares unfavourably with metastases at other sites; isolated soft tissue metastases have a median survival of 50 months (Tampellini *et al*, 1997), bone 33–48 months (Sherry *et al*, 1986) and pleural disease 6–15 months (Johnson *et al*, 1998). Only cerebral metastases confer a worse prognosis with a median survival of only 4 months (Johnson *et al*, 1998). Survival may be prolonged by chemotherapy or endocrine therapy and a small proportion of patients may survive for 5 years (3%) or even 10 years (1%) with these therapies (Sledge and Miller, 1999). Current recommendations are that patients with asymptomatic, oestrogen receptor (ER) positive liver metastases may be treated with endocrine therapy (Buzdar, 2001). Those with symptomatic metastases or ER negative tumours are treated with combination chemotherapy such as FEC (5-fluorouracil, epirubicin and cyclophosphamide) or CMF (cyclophosphamide, methotrexate, 5-fluorouracil) (Fossati *et al*, 1998; Sledge and Miller, 1999). Taxane chemotherapy may be used in anthracycline-resistant disease (Hortobagyi *et al*, 2000). Novel therapies with monoclonal antibodies such as trastuzumab (for c-erb-B2/Her-2 positive breast cancers) are also being used often in combination with taxane chemotherapy (Fournier *et al*, 2000). Chemotherapy will achieve a response in 50–80% of patients and survival may be prolonged (Hortobagyi, 2000). However, the side effects of these agents may be severe and occasionally result in fatal complications.

There have been few studies of surgical treatment of liver metastases from breast cancer, either for attempted cure or debulking. Several studies suggest a small subgroup of patients may gain a survival advantage from surgery and there are reports of rare long-term survivors (Pocard *et al*, 2000; Selzner *et al*, 2000).

The aim of this study was to identify factors that enable prediction of those patients unlikely to survive for prolonged periods, for whom chemotherapy may be of no benefit and impair their remaining quality of life.

Liver metastases may present asymptomatically during a metastatic screen, or may present with upper abdominal fullness, a mass, ascites, jaundice or weight loss (O'Reilly *et al*, 1990). Ultrasound or CT scan usually confirms the diagnosis. Liver function tests are deranged in 92% of patients at presentation with gamma glutamyl transferase (GGT) and alkaline phosphatase being the most commonly elevated enzymes. Overt jaundice is less common (13%) (O'Reilly *et al*, 1990).

Factors adversely affecting prognosis include: jaundice (Hoe *et al*, 1991), deranged liver function tests (Zinser *et al*, 1987; O'Reilly *et al*, 1990), ascites, palpable hepatomegaly (O'Reilly *et al*, 1990), poor performance status and disease confined to the liver (Zinser *et al*, 1987; Hoe *et al*, 1991). The interval between primary presentation and metastatic disease is an important predictor of survival in bone metastases (Sherry *et al*, 1986) but may not be important in liver metastases (O'Reilly *et al*, 1990). The tumour marker CA15-3 is often higher in patients who do poorly, but is not reported to be an independent predictor of survival (Tampellini *et al*, 1997). Carcinoembryonic antigen (CEA) has not been previously studied in this respect although it is recognised as useful in monitoring disease progression (Cheung *et al*, 2000).

The influence of disease pattern, both outside the liver and within the liver has received little attention. Two studies suggest that extrahepatic disease may impair survival (Zinser *et al*, 1987; Hoe *et al*, 1991), but there are no data on the prognostic significance of disease distribution within the liver.

This study has examined the cases of all patients presenting in the last 5 years to a single breast unit with metastatic breast cancer involving the liver at metastatic diagnosis. Survival from the time of metastatic diagnosis was compared with primary disease information, patient characteristics and pattern of metastatic disease in an attempt to establish factors predicting outcome. It is hoped that these prognostic factors may be of benefit in tailoring treatment to avoid toxicity to patients with only a short life expectancy for whom palliative support would be most appropriate.

## MATERIALS AND METHODS

All patients presenting to the Nottingham City Hospital Breast Unit with liver metastases between January 1997 and January 2002 were studied. Upon diagnosis with metastatic disease, full staging was performed including blood tests, (liver function tests (LFTs), full blood count (FBC), erythrocyte sedimentation rate (ESR), CEA and CA15-3), a liver ultrasound or CT scan, chest X-ray and bone scan. Liver biopsy was performed only in cases of diagnostic doubt.

Patients were treated either symptomatically or with endocrine or chemotherapy. Treatment selection was based on the fitness of the patient to tolerate chemotherapy, disease symptoms and the patient's wishes. During treatment, patients were reviewed clinically, radiologically and biochemically every 12 weeks or more frequently during chemotherapy. The UICC criteria were used to assess response (Hayward *et al*, 1977). For the purpose of this analysis, the term ‘responder’ refers to patients experiencing a documented response for a minimum of 6 months on endocrine therapy or 3 months on chemotherapy (British Breast Group, 1974). Responders included complete response, partial response and static disease (Clinical Benefit Rate). Nonresponders included only patients with documented progressive disease. The maximal duration of response was noted.

Tumour marker levels (CA15-3 and CEA, either increasing or decreasing during treatment) were also assessed. Changes in tumour markers alone were never considered sufficient criteria for changing therapy which also required either clinical or radiological confirmation. Once disease progression was confirmed, treatment was changed and the assessment protocol repeated. The cause of death was recorded from the case notes.

The time from metastatic diagnosis to death or the time of analysis was then compared with patient and disease characteristics to determine prognostic significance. Grade was determined using the Nottingham System and classified as grade 1, 2 or 3 (Elston and Ellis, 1991). Oestrogen receptor analysis was by calculation of McCarthy's Histochemical (H) score (McCarty *et al*, 1983), with a score of greater than zero being regarded as ER positive in the metastatic setting. The lymph node stage and the size of the primary tumour in millimetres were also recorded. The Nottingham Prognostic Index (NPI, grade+nodal status+0.2 × primary size in cm) (Haybittle *et al*, 1982) was calculated for each patient at primary presentation.

The Nottingham Secondary Prognostic Index (Robertson *et al*, 1992) was calculated to assess whether this was a useful predictor of outcome when considering a single dominant metastatic site. This was calculated as follows:


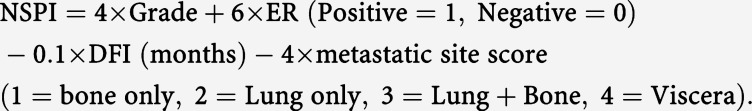


This scoring system is of value in determining prognosis in patients with metastatic disease at different sites. Patients with a score of less than 8 are categorised as good prognosis, 8–16.5 as moderate prognosis and >16.5 as poor prognosis. The score was calculated for each of the patients in this series and compared with survival in months.

Analysis was performed using Kaplan–Meier survival curves with *χ*^2^ analysis or Kruskal–Wallis ANOVA plus Mann–Whitney *U* testing to compare medians between groups. Multivariate analysis was performed using pretreatment variables to determine which factors were independent prognostic factors. Statistical significance was accepted if *P*<0.05. All data are represented as the median plus range.

## RESULTS

### Overall survival

A total of 506 patients have presented to the Nottingham Breast Unit over a 5-year period with metastatic breast cancer to different sites. Of these, 145 were found to have liver metastases at the time of initial presentation (28.7%). Of these 145 patients, 100 have died (69%), with a median survival from time of metastatic diagnosis of 4.23 months (range 0.16–51). There were 40 1-year survivors, six 2-year survivors and one 3-year survivor. No patient has so far survived 5 years in this study group. The median age of the patients was 61 years (range 24–92).

### Characteristics of primary cancer

Of those patients who initially had presented with operable primary breast cancer (*n*=93, 72.1%), the median NPI, at presentation was 5.13 (range 2.4–7). The remainder of patients either presented with inoperable locally advanced primary cancer (*n*=21, 16.3%), or the disease was metastatic at presentation (*n*=15, 11.6%). Oestrogen receptor positivity was found in 38.6% of tumours, 33.1% were ER negative and 28% were ER unknown. The majority of patients had grade 3 cancers at presentation (grade 3: 50.3%, grade 2: 22.1%, grade 1: 0.7%).

Both the ER status and grade had a significant effect on prognosis, with ER-positive patients having a median survival of 7 months (0.4–32) and ER-negative 3.65 months (0.16–51). The grade of tumour had a similar effect with patients with grade 1 or 2 tumours (combined) having a median survival of 6 months (0.7–51) and those with grade 3 tumours median survivals of 3.53 months (0.16–31). The wide ranges of these subgroups, however, mean that clinical application is limited.

The median time from primary to metastatic breast cancer diagnosis was 29 months, (range 0–291 months). This time period had no prognostic significance.

The Nottingham Secondary Prognostic Index did not predict survival, with no significant difference between good, moderate and poor prognostic groups, although a trend for shorter survival was seen in the poor prognostic group with a median survival of 4.13 months (0.16–51) compared with good and moderate groups at 8.45 (0.73–26) and 7.15 (0.57–20), respectively. This probably reflects the heavy weighting of visceral metastases in this scoring system.

### Pattern of metastatic disease

Most patients had metastatic disease at multiple sites, with only 25.5% having liver metastases alone (Table 1

**Table 1:** Distribution patterns of breast cancer metastases within and outside the liver and the effect on survival times

**Metastatic distribution**	**Number (percentage) affected**	**Median survival+(range) (months)**
*Within the liver*		
Single metastasis	28 (19.3)	10.34 (1.1–51)
Multiple non-confluent metastases	84 (57.9)	4.6 (0.16–30)^a^,^b^
Diffuse parenchymal metastases	12 (8.3)	1.85 (0.16–31)^a^
Multiple confluent metastases	22 (15.3)	1.66 (0.16–20)^a^
		
*Outside the liver*		
Liver alone	36 (24.8)	4.97 (0.16–30) NS
Liver and bone	61 (42.1)	3.87 (0.16–51) NS
Liver and lung	34 (23.4)	5.75 (0.16–51) NS
Liver and pleura	24 (16.6)	4.27 (0.7–23) NS
Liver and other^c^	40 (27.6)	4.7 (0.16–32) NS
Liver and multiple sites	43 (29.7)	5.1 (0.3–51) NS

Data shown are the median survival times from the date of diagnosis with liver metastases (plus range). Patients in the site-specific rows may be included in the multiple sites rows.

a Denotes statistical significance compared with survival for single metastasis.

b Denotes statistical significance compared with survival for diffuse and multiple confluent meatastases, *P*<0.05, Kruskal–Wallis ANOVA and Mann–Whitney *U*-test.

c Other metastases include four CNS, two ovarian, 18 with para-aortic, mediastinal or supracalvicular nodes, nine with ascites, one adrenal, one endometrial and two cutaneous. NS=not statistically significant.

). There was no difference in survival between patients with metastatic disease confined to the liver *vs* disease at more than one site (Table 1).

The pattern of disease within the liver was most commonly nonconfluent multiple metastases, with single metastasis the next most frequent pattern and diffuse involvement of the liver parenchyma seen least frequently (Table 1). The pattern of metastases within the liver had a significant effect on prognosis with patients with a single focus within the liver having the best prognosis and confluent multiple metastases the worst (Table 1). The median survival of the 6 patients with a single focus in the liver and no metastatic disease elsewhere was 10.5 months (1.77–26).

### Liver function tests

The majority of patients had deranged liver function tests at presentation (Table 2

**Table 2:** Biochemical parameters influencing survival times with liver metastases from breast cancer

**Biochemical test (normal range+units)**	**Median survival if normal (months)**	**Median survival if abnormal (months)**
ALT <50	6.0 (0.16–51)	2.6 (0.16–25)*
(Up to 50 IU)	*n*=87	*n*=49
Alkaline phosphatase <1000	5.13 (0.16–51)	1.1 (0.16–25)*
(Up to 300iu)	*n*=113	*n*=22
Alkaline phosphatase <500	6.96 (0.17–51)	1.58 (0.16–31)*
(Up to 300 IU)	*n*=89	*n*=46
Albumin <35	7.0 (0.27–51)	2.0 (0.16–27.2)*
(35–45 g l^−1^)	*n*=82	*n*=51
Albumin <30	5.86 (0.16–51)	1.5 (0.16–5.13)*
(35–45 g l^−1^)	*n*=116	*n*=20
Bilirubin >50	4.9 (0.16–51)	0.6 (0.16–1.57)*
(<18 mmol l^−1^)	*n*=131	*n*=6
Bilirubin >20	5.1 (0.16–51)	1.38 (0.16–25)*
(<18 mmol l^−1^)	*n*=117	*n*=18
GGT >250	6.0 (0.16–51)	2.67 (0.16–31)*
(Up to 50 IU)	*n*=90	*n*=44
CEA >1000	4.9 (0.16–51)	1.035 (0.4–5.1)*
(Up to 10 ng ml^−1^)	*n*=127	*n*=8
CEA>10	5.26 (0.16–32)	4.0 (0.16–51) NS
(Up to 10 ng ml^−1^)	*n*=70	*n*=65
CA15-3 >35	6 (0.4–32)	4.2 (0.16–51) NS
(Up to 35 U ml^−1^)	*n*=26	*n*=110

ALT=alanine amino transferase; GGT=gamma glutamyl transferase; CEA=carcinoembryonic antigen. Data shown are the median survival times from the date of diagnosis with liver metastases plus the range in brackets. Thenumber of patients in each group is also shown (*n*).

* Denotes statistical significance, *P*<0.01. NS=not significant.

). The most common abnormality was an elevated GGT (73.1%), followed by raised alkaline phosphatase (60%), low albumin (35.2%), elevated alanine amino transferase (ALT, 33.8%) and serum bilirubin elevation (12.4%). Some of the patients with alkaline phosphatase elevation had concomitant bone metastases, which may have contributed to cases where this was the only abnormal LFT.

**Figure 1:**
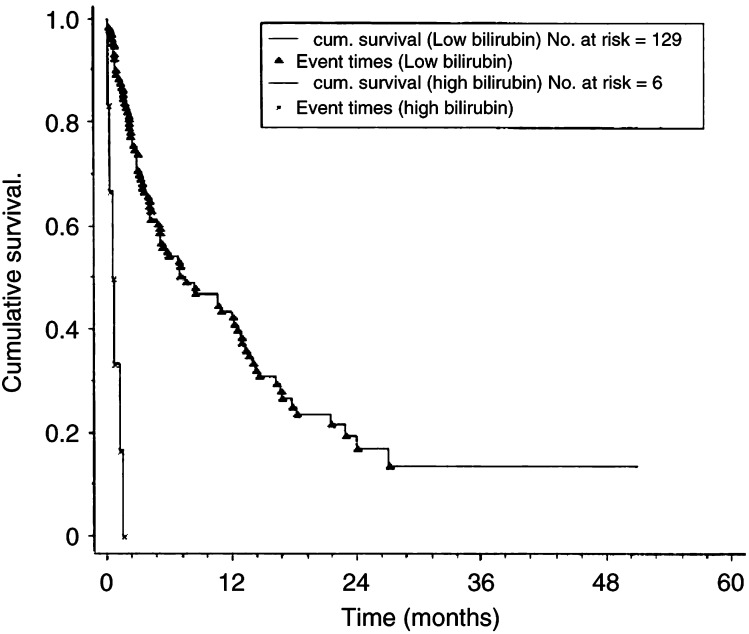
Kaplan–Meier cumulative survival plots for survival with liver metastases according to serum bilirubin level at metastatic diagnosis. Low bilirubin refers to serum concentration of less than 50 *μ*M l^−1^. High bilirubin refers to serum concentration of greater than 50 *μ*M l^−1^. *P*<0.0001, (*χ*^2^ 53.4, 1 df).

Median survivals were compared between patients with normal, mildly abnormal (any deviation from the normal range) or markedly abnormal liver function tests. Both mildly and severely abnormal values were associated with a significantly reduced median survival (Table 2). The most reliable predictor of poor prognosis was a serum bilirubin of over 50, which was associated with a median survival of 0.6 months with no patient surviving more than 1.57 months, (Figure 1

). The next most significant predictor was a serum albumin of less then 30 g dl^−1^ with a median survival of 1.5 months and no patient surviving longer than 5.1 months. For all of the other LFTs, although the median survivals were significantly reduced, the range of survival included patients surviving for over 2 years and are therefore not absolute indicators of poor prognosis.

### Tumour markers

Serum tumour markers were elevated above the normal range (>35 U ml^−1^) in 75.9% for CA15-3 and 44.8% of cases for CEA (>10 ng ml^−1^).

CA15-3 levels were studied at both above and below the normal range and also above 1000 U ml^−1^. Although the median survival suggested a trend for a worse prognosis with higher levels, no statistically significant difference was detected, and therefore CA15-3 cannot be used to estimate prognosis (Table 2).

Carcinoembryonic antigen (CEA) levels were studied at both above and below the normal range and also above 1000 ng ml^−1^. At a cutoff of 10 ng ml^−1^, the normal upper limit, no useful predictive value was found. However, if a level of 1000 ng ml^−1^ was used, median survival was not only dramatically reduced, but the longest survival was only 5.1 months, which suggests that a serum CEA level of greater than 1000 ng ml^−1^ may have useful prognostic implications. However, this factor is of limited value as only eight patients had CEA elevation of this magnitude (Table 2).

### Patient characteristics

In most cases, the liver metastases were asymptomatic at the time of metastatic diagnosis and were detected by a metastatic screen when the patient presented with symptomatic metastases elsewhere (*n*=88, 78.6%). The remainder were symptomatic, presenting with epigastric pain or fullness (*n*=24, 21.4%). Symptomatic liver disease carried a worse prognosis than asymptomatic liver metastases with median survivals of 4.9 (0.16–26) and 9.2 (0.5–51) months, respectively (*P*<0.001). Palpable hepatomegaly was present in only 30 patients (27.3%) but indicated a poor prognosis (median survival of 5.8 *vs* 9.1 months, *P*<0.0004).

The presence of ascites was uncommon (*n*=9, 6.2%) but was a significant predictor of poor prognosis, with a median survival of 0.8 (0.16–8) months *vs* 5 months (0.16–51) if absent (*P*<0.003). In addition, the longest survival with ascites was only 8 months and therefore this parameter may be clinically useful in making treatment decisions.

**Figure 2:**
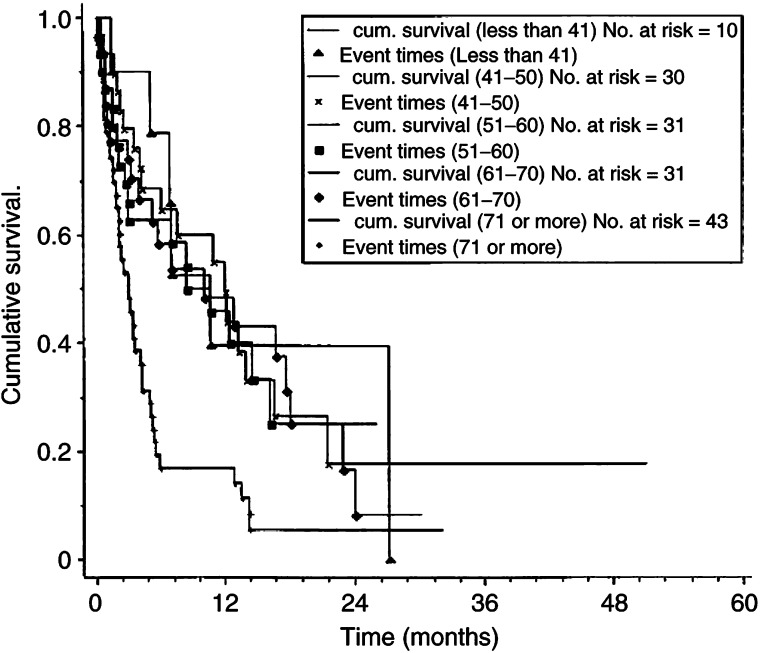
Kaplan–Meier cumulative survival plots for survival with liver metastases by age group. Categories refer to age at metastatic diagnosis. *P*<0.0001 (*χ*^2^ 20.8, 4 df).

Patient's age influenced survival; patients over the age of 70 years fared worse than all other age groups (>70 years of age: median survival 3 months (0.16–32), <70 years: median survival 6.0 (0.16–51), *P*<0.002, Figure 2

). However, because of the wide range, this parameter may not be clinically useful.

### Multivariate analysis

Multivariate analysis was performed to assess which of the patient and disease characteristics (pretreatment) were independent predictors of survival. Only age, a low serum albumin and ER status were found to predict independently poor survival. If age was excluded from the multivariate analysis, grade became independently important.

### Treatment type and response

Complete data on treatment were available on 115 of the 145 patients.

The patient's clinical status, fitness, hormone receptor status and age determined the most appropriate treatment. Multiple different treatments were often given. A total of 13 patients received palliative treatment from the outset. Chemotherapy, between one and four different regimens, was given to 58 patients, often in conjunction with endocrine therapy. Endocrine therapy was given to 60 patients, of whom 39 had endocrine therapy alone. The group receiving endocrine therapy was significantly older than that given chemotherapy with a median age of 75 years for endocrine therapy (range 45–92) *vs* 51 years for the chemotherapy group (range 24–77, *P*<0.00). Only three patients over the age of 70 years received chemotherapy (aged 70, 71 and 77 years).

#### Symptomatic therapy only

Patients in this group were treated with analgesia and/or steroids. The median age was 66 years (range 41–87), which was significantly higher than the chemotherapy-treated group (*P*<0.002). The median survival for this group (*n*=13) was 0.8 months (0.16–4.2).

#### Endocrine therapy

Of the 60 patients in this group, 39 received endocrine therapy alone with 21 also having some form of chemotherapy. Chemotherapy and endocrine therapy were given sequentially rather than concurrently. Confining analysis to patients for whom endocrine therapy was the sole treatment, only six (15.4%) had a response lasting at least 6 months. All of these patients had ER-positive tumours, which tended to be low grade. Of all nonresponders to endocrine treatment (including those who also had chemotherapy, *n*=54), 34 were ER positive, 14 were ER negative and five cases ER unknown. Many patients had several types of endocrine therapy with a total of 80 courses being prescribed. Of the 80 courses, only six responded at 6 months (7.5%, Table 3

**Table 3:** Types and responses achieved with different courses of endocrine or chemotherapy

**Type of therapy**	**Number of courses**	**Progressive disease**	**Static disease**	**Partial response**	**Complete response**
*Endocrine therapy*		*Best response achieved for at least 6 months*			
Anastrazole	29	27 (93)	1 (3.4)	0	1 (3.4)
Tamoxifen	20	17 (85)	2 (10)	1 (5)	0
Anastrazole+					
Goserelin	5	5 (100)	0	0	0
Megestrol acetate	17	17 (100)	0	0	0
Exemestane	5	5 (100)	0	0	0
Fulvestrant	2	2 (100)	0	0	0
Ethinyl oestradiol	2	2 (100)	0	0	0
					
*Chemotherapy*		*Best response achieved for at least 3 months*			
CMF	11	3 (27.3)	4 (36.4)	3 (27.3)	1 (9.1)
Anthracyclin alone	12	5 (41.6)	2 (16.7)	2 (16.7)	3 (25)
Doxorubicin/taxane	8	3 (37.5)	0	3 (37.5)	2 (25)
Taxane alone	8	2 (25)	0	6 (75)	0
CCI	8	2 (25)	3 (37.5)	1 (12.5)	2 (25)
FAC/FEC	27	4 (14.8)	6 (22.2)	13 (48.1)	4 (14.8)
Vinorelbine	2	1 (50)	0	1 (50)	0
Other^a^	6	2 (33.3)	0	4 (66.6)	0

Some patients are included more than once Data shown are patient numbers with percentage in brackets. The number of patients in each group is too small for meaningful statistical analysis. CMF=cyclophosphamide, 5-fluorouracil, methotrexate; FAC/FEC=Fluorouracil, cyclophosphamide and either doxorubicin or epirubicin; CCI=Cell cycle inhibitor. Taxanes included both docetaxel and paclitaxol.

a Other=mitozantrone, trastuzumab plus paclitaxol, CMF+doxorubicin, 5-fluorouracil infusion and 3M (mitomycin, methotrexate and mitozantrone).

).

Direct comparison between endocrine- and chemotherapy-treated patients is not appropriate as patients selected for these treatments differed in age and fitness. However, endocrine treatment, if successful, significantly prolonged life. The six patients responding to endocrine therapy had a median survival of 13.9 months (range 6–26) compared to 2.6 months (range 0.56–32) for nonresponders (*P*<0.0002). The responders had relatively favourable prognostic features such as normal LFTs, single or nonconfluent liver metastatic pattern and little or no extrahepatic metastatic disease.

#### Chemotherapy

A total of 58 patients had chemotherapy, with a total of 82 courses of various types being given. In all, 21 patients also had endocrine therapy but only one responded. No response to chemotherapy was seen in 18 patients (including two who died after one cycle of treatment, and two who only had one cycle due to poor tolerance), 10 patients responded for at least 3 months and 29 responded for at least 6 months. The response rate (3 or 6 months) per treatment course was 60 out of 82 courses (73.2%). A total of 22 patients went on to have two types of chemotherapy, eight had three types and one patient had four types of chemotherapy. The relative survival times of these three groups were 4.6 months for nonresponders (range 1.2–12), 10.23 for 3-month responders (range 4.1–23, *P*<0.003) and 13.23 for 6-month responders (range 6–51, *P*<0.003, Table 3).

Two deaths were related to chemotherapy: one to zoster septicaemia and the other to massive pulmonary embolism.

### Cause of death

The cause of death was recorded in 75 of the 100 deceased patients, although it should be noted that the cause of death recorded in the notes in patients with known metastatic disease may be unreliable. In 73 of the 75 cases (97%), carcinomatosis was either the primary cause (64 cases) or a significant contributing cause of death (nine cases). Two patients died as a result of chemotherapy (see above). Six deaths were contributed to by peptic ulcer disease (haemorrhage *n*=2, perforation *n*=4), often in patients on NSAIDs or steroids or both.

Cerebral metastases were seen in 16 patients. In four, they were diagnosed at metastatic presentation. In 12, they were diagnosed during or after chemotherapy despite a good response in extracerebral sites. The median survival of patients with cerebral metastases overall was good from the time of diagnosis of liver metastases being 9.5 (0.8–27.2) months, but median survival from the time of diagnosis of cerebral disease was only 1.25 (0.5–18.1) months, despite treatment with cranial irradiation and steroids.

### Factors not influencing survival

No prognostic significance was found to be attached to the metastases-free interval, the presence of other metastatic sites (Table 1), the histological subtype, the nodal stage at presentation, the Nottingham Prognostic Index (primary) or the presence or absence of vascular invasion.

## DISCUSSION

Liver metastases from breast cancer have a poor prognosis, with a median survival in this study of only 4.23 months. This is similar to, or better than, the findings of other published series (Patanaphan *et al*, 1988; O'Reilly *et al*, 1990; Hoe *et al*, 1991). Those patients receiving chemotherapy had a relatively good overall prognosis, with responders surviving for a median of 13 months. Those responding to endocrine therapy (*n*=6) fared even better with a median survival of 13.9 months. It must be remembered, however, that patients who received chemotherapy were a selected group with a lower median age and better general health than the non-chemotherapy-treated group. For example, only one of the patients presenting with evidence of liver failure (i.e. serum bilirubin of over 50, palpable hepatomegaly, ascites, hypoalbuminaemia) received chemotherapy but the patient died during the first cycle. Most such patients were either treated palliatively or with endocrine therapy. Endocrine therapy was seldom effective and of the six patients who responded, all were ER positive. This is comparable with the response rate quoted by O'Reilly *et al* (1990) but lower than the overall response rate of metastatic disease at other sites of 65% (Buzdar, 2001). It is disappointing that the response rate has not increased despite the current availability of an increased range of endocrine therapies. Patients were often treated with endocrine therapy if they were unfit for chemotherapy and in the group of 39 patients receiving endocrine therapy, 29 were over the age of 70 years and, of these, 14 had an albumin of less than 35 g dl^−1^ at presentation. This may explain much of the poor performance of endocrine treatment in this study.

The older age group of patients did significantly worse than the under 70-year age group. Mortality differences with increasing age in primary breast cancer patient may be due to death from comorbid conditions (Yancik *et al*, 2001), but the breast cancer-specific mortality also increases with age when metastatic disease is present (Yancik *et al*, 1989). This is in agreement with our data, where cause of death was almost always due to carcinomatosis, even in the elderly. Treatment practice differed with age, with 56% (55 out of 98) of the under 70s receiving chemotherapy, but only 6.4% (three out of 47) of the over 70 s. Chemotherapy tolerance may be reduced in the elderly (Walsh *et al*, 1989) and reduced renal function may necessitate dosage reduction; this may reduce toxicity but compromises efficacy, (Gelman and Taylor, 1984). Despite this, there are reports of successful use of the chemotherapy in the elderly (Walsh *et al*, 1989). In view of the poor endocrine response rates for liver metastases, should we be exploring chemotherapy strategies for the elderly?

Peptic ulcer disease complications contributed to death in six cases, all of whom were taking nonsteroidal anti-inflammatory drugs or steroids. This highlights the need for gastric mucosal protection in this high-risk group.

Cerebral metastases, after a good extracerebral response to chemotherapy, were relatively common. The blood–brain barrier excludes most chemotherapeutic agents from the CNS, which therefore acts as a sanctuary site for metastases (Carey *et al*, 2001). Prevention of this problem is difficult. Prophylactic cranial irradiation might slow down or prevent the development of cerebral metastases. However, there is a high risk of cognitive impairment (DeAngelis *et al*, 1989). In addition, only 11% of patients developed symptomatic cerebral metastases, which would not justify widespread use, especially in view of the absence of proven therapeutic benefit.

With regard to prediction of likely survival from patient and disease characteristics, it has been shown that liver function test derangement, of almost any degree, is associated with a poor outcome (Zinser *et al*, 1987; O'Reilly *et al*, 1990). However, the range of survival reveals that, although the median survivals are lower with deranged LFTs, there are still patients who survive for prolonged periods and therefore in most cases no definite prognostic implications can be derived. However, in the case of patients with both a low albumin (<30 g dl^−1^) and a high bilirubin (>50 *μ*mol l^−1^), denoting the onset of liver failure, the median survival was only 0.6 months (range 0.16–1.57). In the presence of these findings, therefore, there is a worse prognosis predictable at the individual patient level. The presence of ascites has a similarly poor outlook.

Very little prognostic information can be gained from the characteristics and stage of the primary tumour in patients with liver metastases. The only prognostic factors were the grade and the ER status of the primary tumour, although once again the range of survival is such that no useful information can be derived on an individual patient basis to facilitate therapeutic choices.

The occurrence of metastases at sites outside the liver had no effect on the outcome for the patient, suggesting that liver metastases are pre-eminent in causing the patient's death. The occurrence of cerebral metastases may confer a worse prognosis, but very few patients had these at the time of metastatic diagnosis and therefore no statistical effect could be demonstrated.

The pattern of metastatic disease within the liver has prognostic implications; patients with single metastasis have the best long-term survival and multiple confluent and diffuse metastases have the worst outcome. Of the single liver metastasis group, only six had no evidence of metastatic disease elsewhere at the time of presentation and one of these had inoperable primary cancer precluding curative liver surgery. The five potential surgical candidates had a median survival of 14 months (range 6–26) with three still living. At present, it is not routine practice to perform liver resection for breast metastases due to the frequent coexistence of metastatic disease elsewhere, the generally poor prognosis and the magnitude of the surgery. There have, however, been reports of long-term survival in carefully selected patients after liver resection for metastases from breast cancer (Pocard *et al*, 2000; Selzner *et al*, 2000).

Tumour markers are used for the monitoring of metastatic disease during treatment (Tampellini *et al*, 1997; Cheung *et al*, 2000) but have limitations as a prognostic tool at the diagnosis of metastatic breast cancer. CA15-3 was elevated above normal in 75.9% of patients at metastatic diagnosis, which is in agreement with other studies (Tampellini *et al*, 1997). The level of elevation could not be used to predict survival, however, even when the analytical cutoff was very high (>1000 U ml^−1^). Carcinoembryonic antigen was more useful. This marker was elevated above normal in 44.8% of patients, in agreement with other data for metastatic breast cancer (Mughal *et al*, 1983). Mild elevation carried no prognostic significance. However, when very high (i.e. above 1000 ng ml^−1^), patients did badly, with a median survival of 1 month. The range was also narrow, with the longest survival being for only 5.1 months, which means that this may be used on an individual patient basis to predict poor outcome.

In summary, this study has demonstrated that several factors may be useful in predicting duration of survival for patients with liver metastases from breast cancer. Of these, the factors carrying the most ominous implications are those denoting the presence of liver failure, that is, a low serum albumin, elevated bilirubin and the presence of ascites. Many other features have a significant impact on survival (disease distribution within the liver, patient age and the grade and ER status of the tumour) but, due to the wide range of survivals within these groups, these values have little use on an individual patient basis. In terms of the type of treatment and response, patients who are given symptomatic treatment survive very poorly, as expected, although this is likely to represent both the treatment and the nature of the patient and their disease. Patients who did best were those responding to chemotherapy and endocrine therapy, in whom survival of several years was sometimes achieved and this should be a source of optimism for both patients and doctors as therapies continue to improve.
